# Mechanochemical Activation of Superradiance in Paramagnetic Polymer Composites

**DOI:** 10.3390/ma16031297

**Published:** 2023-02-02

**Authors:** Aleksey I. Aleksandrov, Vitaliy G. Shevchenko

**Affiliations:** Enikolopov Institute of Synthetic Polymeric Material Russian Academy of Sciences, Moscow 117393, Russia

**Keywords:** mechanochemistry, rheological explosion, superradiance

## Abstract

The review examines the effect of radio-frequency superradiance during pulsed mechanochemical activation of polymer composites under high pressure. Mechanochemical activation is implemented in three modes: (a) rheological explosion of polymer composite under rapid uniaxial compression, when an elastic wave pulse occurs in a polymer composite sample and implements the physico-chemical transformations leading to the occurrence of a superradiance pulse; (b) parametric mode, when an elastic wave pulse is introduced from the outside through a waveguide into a composite sample; (c) the mode of rapid pressure release, which also leads to the occurrence of a superradiance pulse. Paramagnetic polymer composites—namely polystyrene–binuclear clusters Co(QH)_2_–O–Co(QH)_2_ or Mn(QH)_2_–O–Mn(QH)_2_, where QH is a ligand based on QH_2_–3,6-di-tert-butylpyrocatechin)—are considered as objects implementing such processes. These binuclear clusters exhibit the Dzyaloshinskii–Moriya effect, and polymer composites based on them exhibit multiferroic properties. A composite of a molecular magnet in polystyrene matrix (Eu(III)(SQ)_3_·bipy complex with four unpaired electrons on Eu(III) and on SQ ligands; SQ is 3,6-di-tert-butylquinolate paramagnetic ligand) is also considered. The binuclear clusters and europium complexes form 2D nano-objects in the polymer matrix with a diameter of 50–100 nm and a thickness of ~ 1–2 nm. The review considers the formalisms of Dicke, Lorentz, Landau–Lifshitz–Blombergen and Havriliak–Negami equations, which make it possible to conduct a time–frequency analysis of these processes, to obtain data on the relaxation processes of spin and charge density in objects responsible for the process of radio-frequency superradiation. It is also shown that the analysis of electron spin resonance data allows us to provide a probable quantum chemical scheme for the implementation of the radio-frequency superradiance process. The phenomenon of superradiation has a great deal of potential in such areas as energy-saving technologies, wireless power transmission and storage devices. The technique of studying fast mechanochemical processes considered in the review allows us to investigate the mechanisms of interaction of magnetic and electrical subsystems in multiferroics and molecular magnets, which expands the scientific base for the creation of new functional materials and enables the solving of related problems of condensed matter physics.

## 1. Introduction

Three phenomena—namely rheological explosion (RE), superradiance (SR) and weak ferromagnetism—have been known for a long time. A new direction in mechanochemistry—pulse mechanochemistry—has been developing quite successfully over the past 30 years [1,2,3,4,5,6]. This review deals with the interaction of these fields of science, which led to the discovery of the phenomenon of radio-frequency SR during pulsed mechanical activation. We briefly discuss the definition of the concepts of RE, SR, weak ferromagnetism and pulsed mechanochemistry.

RE was first discovered by P.W. Bridgman [1]. This phenomenon is associated with the ultrafast destruction of solids under uniaxial compression—intense pulses of elastic waves, charge density transfer, electromagnetic radiation and rapid solid-phase chemical reactions, which cannot be explained by classical concepts of thermal activation [2,3,4,5,7,8,9,10,11]. It was shown in [5,7,8,9,10,11] that substances such as ammonium bichromate and oxalic acid crystallohydrate (H_2_X_2_O_4_·H_2_O) (at 300 K) and ammonium–water ice (at 100–250 K) exhibit explosive instability under rapid uniaxial compression at high pressure, i.e., RE is observed in these objects. In ref. [8], the effect of the hypersensitive response of compressible (from 0.1 to 1.0 GPa) crystallohydrates was discovered and investigated. When exposed to very weak electric fields with a frequency of 20–50 Hz and amplitude of Δ*E* = 65 V, it was found that such fields lower the pressure threshold for the onset of RE from 1.6 to 0.6 GPa, i.e., almost three times lower. However, these objects are quite unstable from an experimental point of view and inconvenient for practical use. It is obvious that to study the RE effect, it is most convenient to use more stable materials, such as polymer composites, available in significant weight quantities. The works of N.S. Enikolopov and his students [4,9,10,11] were devoted to these issues, where physico-chemical transformations in processes such as RE were studied, and it was found that RE in a chemically active medium can initiate a wave of self-propagating chemical reactions. In [4,10], it was suggested that both mechanochemical processes in solids and self-propagating chemical processes under the influence of elastic wave pulses from RE are initiated by dipole clusters that link acoustic and optical phonons, which initiate the occurrence of chemical transformations. The mechanism proposed in [4,11] is in good agreement with the concepts of phonon [12] and exciton dislocation [13], destruction of solids and ionic crystals, as well as with the ideas of the connection between the front of solid-phase reactions with internal mechanical stresses and nonlinear waves [14,15]. In further studies, attention was mainly paid to the study of the nature of chemical transformations under the influence of elastic wave pulses (EW) from a RE on polymer and organoelement compounds.

Let us briefly dwell on the essence of SR phenomena. The SR effect is a process of coherent spontaneous radiation, which was first indicated in 1954 in the theoretical work of R. Dicke [16]. The prototype of SR is laser radiation, but the process of SR generation is fundamentally different from laser generation. In the latter, the transition of excited atoms to a lower energy state, as a result of induced radiative transitions, occurs in each atom individually, independently of other atoms—i.e., excited atoms and molecules are not in collective interaction. The process of superradiation is realized collectively by the phased excited centers—that is, the removal of excitation in each excited atom does not occur independently of other atoms (molecules), but collectively. SR is the process of electromagnetic radiation of the medium as a single phased ensemble (macrodipole) consisting of microdipoles covered by collective interaction through a cooperative communication wave. The intensity of the SR pulse is *W_C_~N*^2^; *N* is the total number of excitation centers, and *W* is the intensity of the SR pulse. The radiation intensity in lasers is inversely proportional to *N*, i.e., *W_L_~N*. The coupling coefficient (*ω_C_*)^2^, a measure of the strength of the exchange-wave process, which guarantees the collective phase of the oscillations of all microdipoles in the superexcited medium, is used to describe the collective dynamics of the superradiant state. In a specific volume of the medium near the natural resonant frequency *ω*_0_ of the excited medium, which corresponds to one of the levels of the excited system, a cooperative wave with a frequency of *ω_C_* and modulated by an electric wave forms a standing wave. Energy is transferred from the electric field wave into the polarization wave in this instance due to polariton resonance. All of the medium’s microdipoles are covered by polarization waves when polarized at a resonant frequency, creating a single phased macrodipole. For all excitation centers (excited atoms and molecules), the energy of the polarization field and the phases of the polarization waves become uniform. Common energy levels define the collectivized superradiant state. The classical Maxwell equations (Equations (1) and (2)), the equations for the average polarization P (Equations (3) and (4)) by Dicke all describe the radiation resulting from transitions between these levels in the macroscopic approximation [16,17,18].

(1) $$rot\vec{E}=-\frac{1}{c}\frac{\partial \vec{B}}{\partial t}\;$$

(2) $$rot\vec{B}=-\frac{1}{c}\frac{\partial (\vec{E}+4\pi \vec{P})}{\partial t}+\frac{4\pi \sigma }{c}\vec{E}$$

(3) $$\frac{\partial^{2}\vec{P}}{\partial t^{2}}+2T_{2}^{-1}\frac{\partial \vec{P}}{\partial t}+\omega_{0}^{2}\vec{P}=\frac{\omega_{c}^{2}\vec{E}}{4\pi }\;$$

(4) $$\frac{d\langle R_{3}\rangle }{dt}=\frac{1}{T_{1}}\left(\frac{N^{2}}{2}-\frac{\langle R_{3}\rangle {}^{2}}{2}+N+\langle R_{3}\rangle \right)$$

Here, *T*_1_ is the time of energy relaxation; *T*_2_ is the time of phase relaxation of active centers.

(5) $$\omega_{c}^{2}=-\frac{8\pi d^{2}\langle R_{3}\rangle \omega_{0}}{\hbar }$$

*d* is the dipole moment of the transition of atoms or molecules at the resonant frequency *ω*_0_. $\langle R_{3}\rangle$ is the average value of the difference in the population levels in a single volume of the medium containing *N* excitable atoms and *n* excited atoms. I.e., $\langle R_{3}\rangle$ = *n* − (*N* − *n*) = 2*n* − *N*. If the right part of Equation (4) is rewritten as $\frac{1}{2}\;$ [(*N* + 1)^2^ − ($\langle R_{3}\rangle$ − 1)^2^], then

(6) $$\langle R_{3}\rangle =1-\left(N+1\right)\mathrm{th}\left[\frac{N+1}{2T}\left(t-t_{0}\right)\right]$$

For $\langle R_{3}\left(t=0\right)\rangle =N$, we have *t*_0_ =$\frac{T_{1}}{N+1}lnN$ and obtain the power of electromagnetic radiation (radiation intensity) as a function of time:

(7) $$I\left(t\right)=I_{0}N^{2}sech^{2}\left[\frac{1}{2\tau_{c}}\left(t-t_{0}\right)\right]$$

*I*(*t*) describes the SR pulse, its power *I_max_*(*t* = *t*_0_) reaching a maximum at *t* = *t*_0_, and *I*_0_ is the radiation intensity of one particle from an ensemble of *N* particles. The maximum value is proportional to *N*^2^, *t*_0_ is the pulse delay time (*t*_0_ = 2*τ_c_lnN*) and *τ_c_* is the pulse width at half–height (*2τ_c_* is the pulse duration). Equations (1)–(7) describe only quantities averaged over an ensemble or over a large volume (macroscopic quantity), and do not directly include quantum processes that are of decisive importance when collective phased correlations are induced at the initial moment of energy pumping. The SR effect occurs in macroscopic samples at a sufficiently high concentration of *N*.

The phenomenon of weak ferromagnetism was experimentally discovered by L. Neel in α-Fe_2_O_3_ [19]. The thermodynamic theory of this phenomenon was developed by I. E. Dzyaloshinskii [20], and the microscopic theory was developed by T. Moriya [21]. In 1959–1961, based on ideas about the phenomenon of weak ferromagnetism, it was theoretically substantiated and experimentally confirmed that substances with weak ferromagnetism can exhibit a magnetoelectric effect (ME) [22]. The essence of ME effect is that when substances are exposed to a magnetic (or electric) field, an electric (or magnetic) response occurs in them, respectively. Later it was found that both linear and nonlinear ME effects are observed in crystals with weak ferromagnetism, which are most conveniently observed in the dynamic mode by acting on the substance with variable fields *E* or *H* of different frequencies [23,24]. The term multiferroics has been applied to three substances that exhibit a direct and reverse ME effect. For systems in which the multiferroic effect is caused by pairs of non-collinear spins, the intrinsic electric dipole moment *P* is associated with a pair of magnetic atoms with inclined spins S*_a_* and S*_b_* and it is written as **P ∝ r × (S*a* × S*b*)**, where *r* is the relative position of the atoms [25]. Since *r* changes in time, the polarization vectors *P* and the magnetic moments of the system *M*, which are associated with the spin ensemble, change accordingly. The dynamic spectra of such systems are described in the framework of the Lorentz model for a harmonic oscillator with attenuation [26]:

(8) $$\ddot{\vec{r}}+2b\dot{\vec{r}}+\omega_{0}^{2}\vec{r}=\frac{q}{m}\vec{E}$$

(9) $$E\left(t\right)=E_{0}e^{-i\omega t}$$

According to electrodynamics, forced oscillations of charged particles lead to polarization of the medium, which for non-interacting similar particles is described by the polarization vector $\vec{P}$ equal to *N*$\vec{p}$, where *N* is the concentration of particles, and $\vec{p}$ = $q\vec{r}$ is the dipole moment of the particle. Then, Equation (8) can be rewritten as:

(10) $$\ddot{\vec{p}}+\gamma \dot{\vec{p}}+\omega_{0}^{2}\vec{p}=\varepsilon_{0}\omega_{R}^{2}E\left(t\right)$$

where $\omega_{R}^{2}=\frac{e^{2}n_{0}}{\varepsilon_{0}m}$ and *n*_0_ is concentration of oscillators. This equation coincides with Equation (3), which is used to describe the SR process, within the framework of the polarization model, taking into account that *γ* = 2/*T*_2_, while $\frac{\omega_{c}^{2}}{4\pi }=\varepsilon_{0}\omega_{R}^{2}$.

Equation (10) is used to analyze the spectra of resonant dielectric losses. Its solution is

(11) $$\varepsilon^{*}\left(\omega \right)=\varepsilon_{\infty }+\frac{\left(\varepsilon_{S}-\varepsilon_{\infty }\right)\omega_{R}^{2}}{\omega_{R}^{2}-\omega^{2}+2j\alpha \omega }$$

Accordingly, the formulas for the imaginary and real components are:

(12) $$\varepsilon^{\prime }\left(\omega \right)=\varepsilon_{\infty }+\frac{\left(\varepsilon_{S}-\varepsilon_{\infty }\right)\omega_{R}^{2}\left(\omega_{R}^{2}-\omega^{2}\right)}{{\left(\omega_{R}^{2}-\omega^{2}\right)}^{2}+4\alpha^{2}\omega^{2}}\;\varepsilon^{\prime \prime }\left(\omega \right)=\frac{2\left(\varepsilon_{S}-\varepsilon_{\infty }\right)\alpha \omega_{R}^{2}\omega }{{\left(\omega_{R}^{2}-\omega^{2}\right)}^{2}+4\alpha^{2}\omega^{2}}$$

*ω* = 2*πf* is angular frequency, *ω_R_* is the characteristic frequency of natural oscillations of charges in a given volume and *α* is the attenuation coefficient. It follows from the above that the radiation spectra, i.e., the bands of radio frequency radiation during a RE, can be analyzed by dielectric spectroscopy methods.

Accordingly, if these processes take place in systems exhibiting the Dzyaloshinskii–Moriya effect, i.e., having weak ferromagnetism, for which polarization processes are caused by a system of non-collinear spins, then the Landau–Lifshitz equation modified by Blomberg (LLB Equation (12)) is applicable to such systems [27,28].

(13) $$\frac{dM}{dt}=-\gamma M\times H+\omega_{r}\left[\chi_{0}H-M\right]$$

where $\chi_{0}=\frac{M_{0}}{H_{0}}$ is static permeability and *ω_r_* is relaxation frequency. The reverse value *τ_r_* = $\frac{1}{\omega_{r}}$ is relaxation time. For the LLB equation, the solution is:

(14) $$\chi_{\alpha }=\chi_{0}\frac{\omega \omega_{H}}{\omega_{res}^{2}-\omega^{2}+2i\omega \omega_{r}}$$

(15) $${\chi^{\prime }}_{\alpha }=\chi_{0}\frac{\omega \omega_{H}\left(\omega_{res}^{2}-\omega^{2}\right)}{{\left(\omega_{res}^{2}-\omega^{2}\right)}^{2}+4\omega^{2}\omega_{r}^{2}}\;{\chi^{\prime \prime }}_{\alpha }=\chi_{0}\frac{2\omega^{2}\omega_{H}\omega_{r}}{{\left(\omega_{res}^{2}-\omega^{2}\right)}^{2}+4\omega^{2}\omega_{r}^{2}}$$

Comparing the systems of Equations (10), (11) and (13)–(15), it is easily found that $\omega_{r}^{2}=\alpha^{2}$ and $\omega_{R}^{2}=\omega_{pe3}^{2}$. It follows from this that a system with the Dzyaloshinskii–Moriya effect, in which radio-frequency SR is observed during a RE, can be analyzed on the basis of the Equations (6), (10), (11) and (13)–(15).

The increased interest in solid-phase chemical processes under mechanical action is apparently recent, which is due to the possibility of creating environmentally friendly technologies for the synthesis of target products. In terms of fundamental scientific research, mechanochemistry studies the effect of deformation on the reactivity of solids [3]. In mechanochemical experiments, the relationship between the type of mechanical energy and chemical transformations of matter is studied. For this purpose, mixing in a mortar or extruders is used, as well as compression using Bridgman anvils that combine high-pressure and shear deformations [2,29] and shock effects in mills of various designs, disintegrators, shock devices and, finally, in shock waves [30,31]. During the mechanochemical action on the substance, various processes occur that lead to the transformation of elastic energy into the energy of vibrational and electronic excitation, ionization, and the rupture of chemical bonds. Such an effect, depending on its energy, is capable of realizing a wide range of phenomena from local heating in a small volume to the emission of high-energy electrons from the region where mechanochemical reaction occurs [3].

All methods of mechanochemical action on a substance differ in such parameters as the power of the action (mechanical energy supplied to the substance per second) and the dose of the action (the total amount of mechanical energy injected). For example, the simplest grinding of a substance in a mortar is characterized by a power of up to 100 W/g and a dose of up to 10^4^ J/g. The processing of a substance on Bridgeman anvils has a power of up to 10^3^ W/g and a dose of up to 10^5^ J/g, and the impact of a shock wave has a power of up to 10^10^ W/g and a dose of up to 10^4^ J/g [32].

On the other hand, different mechanochemical processes require different dose and impact energies. Thus, crushing requires 0.01 MJ/mol, grinding and fine grinding 0.1 MJ/mol, organic synthesis 1 MJ/mol, and inorganic synthesis and mechanical fusion up to 100 MJ/mol [32]. At the same time, the impact dose should also differ, since it is necessary to overcome the threshold level beyond which such a reaction can occur. An example of such a threshold is the synthesis of diamonds [33] from graphite under the action of a shock wave (the impact power is maximum). Such synthesis is not possible with a lower impact power, for example, in a mill.

However, most experimental approaches have two significant drawbacks. First, the effects on the solid matrix cannot be accurately quantified and, as a result, it is impossible to estimate the yields of mechanochemical reactions. Second, in all the devices described above, it is impossible to separate thermal and non-thermal processes (associated only with deformations). It is still not clear [2,3,10,11] what happens to the substance at the stage when, after mechanical action, elastic energy is not instantaneously and incompletely converted into Joule heat. That is why, unlike photochemistry and radiation chemistry, where fast processes involving high-energy intermediates of chemical reactions have been studied in sufficient detail to date, in mechanochemistry, chemical transformation mechanisms are investigated by analyzing the final products that can also occur during thermal exposure. In the review [3], it was noted that in order to study the intermediates of mechanochemical reactions, it is necessary to supply mechanical energy faster than the rate at which it turns into Joule heat and quickly investigate the resulting products, i.e., actually implement experiments similar to pulsed photolysis [34] or radiolysis [35,36].

That is why the school of N.S. Enikolopov developed a new experimental approach, in which the impact of low-intensity elastic wave pulses (further referred to as EWP in the text) was combined with thermal and matrix stabilization of intermediate products [4,11,37,38,39].

## 2. Mechanochemical Synthesis of Organoelement Radicals, Biradicals, Triradicals and Coordination Polymers under the Action of Elastic Wave Pulses

At the beginning of this research, the samples were simultaneously subjected to uniaxial compression in a closed volume between two steel waveguides, and to the effects of EWP. These pulses were generated during explosive destruction under pressure (during RE) of polypropylene, polyethylene or polystyrene plates, and then the pulses were fed through a steel waveguide into the samples.

In such experiments, the characteristic of the elastic wave pulse power was the value of pressure *P_RE_* at which the RE occurs, and the upper bound of the absorbed energy was estimated from the formula $E=V\frac{P_{RE}^{2}}{2G_{Y}}$, where *G_Y_* is Young’s modulus and *V* is the volume of the steel waveguide. Substituting the characteristic parameters of the experiment (*V* = 3.17 × 10^−6^ m^3^, *P_RE_* = 5–30 kbar), as well as the known value of *G_Y_* = 2 × 10^11^ Pa, we obtain E ≈ 2–72 J. Since the mass of the sample is usually equal to 80 mg, we find that when exposed to EWP, the dose of a single exposure is 25–900 J/g, and the power is 1.25 × 10^6^–4.5 × 10^7^ W/g.

**Figure 1:**
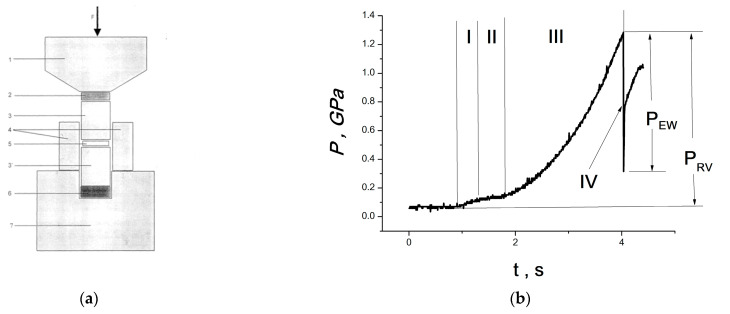

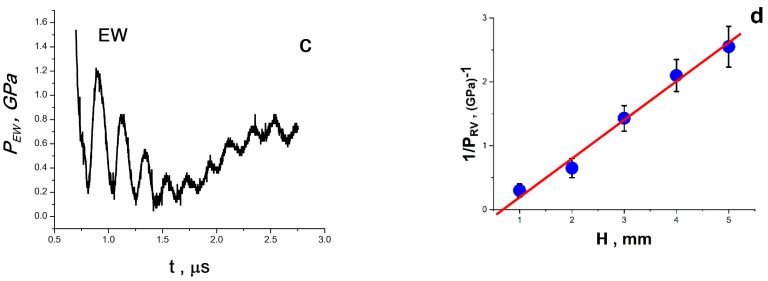
(**a**) Diagram of the device for the action of EWP: 1-Bridgman anvil; 2-polymer plate; 3, 3′-steel waveguides; 4-steel cage; 5-sample; 6-piezoelectric sensor; 7-steel frame. (**b**) Diagram of the pressure build-up on the sample. (**c**) Waveform of the elastic wave pulse excited by a RE. (**d**) The dependence of the RE pressure on the thickness of the polymer plate (PP) is given in coordinates 1/*P_RE_*-*H*.

The following cycle of research was carried out with the installation, which is shown in Figure 1a. The EWP was excited in a steel waveguide (3 in Figure 1a) by a RE occurring during uniaxial compression of polymer plates (polyethylene, polypropylene, polystyrene). Then, the pulses of elastic waves entered the sample. It can be seen from Figure 1a that the sample was simultaneously subjected to uniaxial compression in a closed volume, in a steel cage between steel waveguides (3 and 3′), and to the action of EWP.

Figure 1b shows a diagram of the pressure build-up on the sample, the form of which is determined by the elastic properties of the polymer plates generating EWP. The pressure increases in several stages: the stage of unsteady creep of the polymer (I), steady creep (II), accelerated creep and destruction (III) and, finally, an explosion under pressure, RE (IV). The pressure at which the RE occurs (*P_RE_*) depends on the thickness of the polymer plate *H* according to the law *P_RE_*~*b*/*H*, (Figure 1), where coefficient *b* depends on the type of polymer. By changing the thickness of the polymer plate and its type (polyethylene, polystyrene, etc.), *P_RE_* can be varied in a wide range, from 0.5 to 3.0 GPa. Moreover, the thickness of the sample can be adjusted, so that it is completely clamped between steel waveguides; that is, there is no explosion of the sample itself, but only the effect of EWP is realized. The first three stages last 4–7 s (depending on the thickness of the plate and the type of polymer); the fourth (RE) lasts several microseconds (~10 microseconds). At the same time, an elastic wave is excited in the steel waveguide (3, Figure 1a). It was recorded using a planar piezoelectric sensor (6, Figure 1a) and is shown in Figure 1c. It can be seen that the first wave front is unloading, and then follows the oscillatory process of elastic wave action.

This method was used to study chemical processes in polycrystalline mixtures of 3,6-di-tert-butyl-quinone (Q) and 3,6-di-tert-butyl-pyrocatechin (QH_2_), as well as the solid-phase synthesis of metal-containing mono–, bi- and triradicals under the influence of EWP on systems M–Q, M_x_O_y_–Q (QH_2_, Q+QH_2_) (where M = Zn, Cd, Al, Ga, In, Tl, Sn, Pb, Bi, Ti, Zr, Ge), and (Cr, Mo, W)CO_6_, Mn_2_(CO)_10_–Q. Mechanochemical yields were found for the obtained products—radical pairs and mono-, bi- and triradicals of organoelement compounds; the processes of the synthesis of ultrafine metal particles (iron, cobalt, nickel) during solid-phase oxidation–reduction reactions in a polymer matrix have also been studied [37,38]. The mechanochemical synthesis of coordination polymers under the action of EWP on a polymer matrix containing Cr, Nb, Mn complexes with ligands based on spatially hindered phenols was investigated; additionally, the synthesis of polymer composites with either a giant magnetic resistive effect or multiferroic properties was carried out [6,40,41,42,43,44]. This research cycle established that when EWP acts on solid organic substances, electron and proton transfer processes are realized; competing reactions occur in the presence of electron acceptors (for example, transition metals).

**Figure 2:**
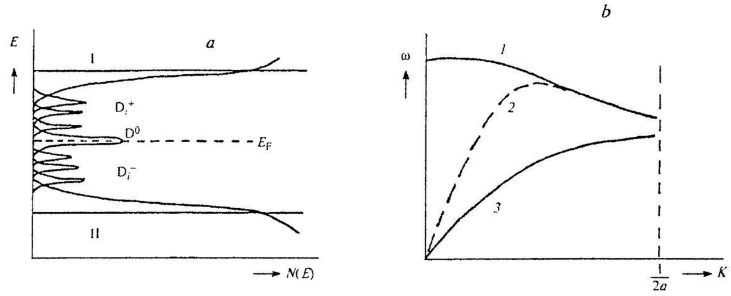
(**a**) A diagram of the density of defective states in an amorphous sample arising under the action of EWP. D_i_^+^ and D_i_^−^ are sets of charged defective states; D^0^ represents metastable molecules. (**b**) typical dispersion curves for the optical (1), soft (2) and acoustic (3) branches of the oscillation spectrum when they overlap (*ω* is the frequency, *a* is the lattice constant, *k* is the wave number).

It was found that from the point of view of the band theory of solids [35], amorphization is associated with the “blurring” of the edges of the conduction and valence bands, as well as with the formation of a set of subzones in the band gap, corresponding to ensembles of negatively (D^−^) and positively (D^+^) charged defects of different dimensions. A diagram of this possible process is shown in Figure 2a. The appearance of blurred subzones contributes to the free transfer of charge density over an amorphous polycrystalline substance. From a chemical point of view, this causes the excitation (activation) of molecules, the formation of free e^-^_K_ and H^+^, charge dipoles on individual molecules or on clusters of these molecules due to uncompensated charges, as well as the appearance of electromagnetic quanta during the deactivation of molecules. The occurrence of dipole formations ensures the connection of mechanical and electrical vibrations and provides overlap (fusion) of acoustic and optical phonon modes in the occurrence of the so-called soft mode (Figure 2b); this phenomenon is characteristic of a wide class of substances [45].

These effects lead, possibly, to the appearance of quasiparticles associated with local vibrations of the lattice of a solid body [46,47]—polaritons and polarons, which almost completely absorb the energy of electromagnetic radiation (ER) quanta [38]. When the critical concentration of dipoles, e^-^_K_ and ER quanta is reached, a self-induced correlation occurs between the dipole moments. All this can be compared with the well-studied processes of SR at optical and hypersonic frequencies [47]. Thus, in [4], the concept of a dipole linking elastic vibrations and electromagnetic waves in a solid was introduced into the scheme of mechanochemical reactions carried out under the influence of EWP, i.e., an electromagnetic–mechanical dipole (EMD). This process follows the equation *ω* = *V_EW_*/*λ_EW_* = *c*/*λ_EMW_*, where *ω* is the dipole oscillation frequency, *V_EW_* and *c* are elastic and electromagnetic wave velocities and *λ_EW_* and *λ_EMW_* are elastic and electromagnetic wavelengths emitted by the dipole. In addition, it was shown in [4] that the introduction of quasiparticles, such as polaritons, into the scheme of mechanochemical processes, their energy consisting partly of electromagnetic energy and partly of the energy of elastic excitation of the medium (polaritons are formed as a result of the interaction of photons with optical phonons, excitons and magnons), allows us to understand where in mechanochemical processes is the missing Joule heat—that is, how the mechanochemical and thermal processes differ from each other.

The concept of an electromagnetic–mechanical dipole, in addition to the previously proposed “convection” model of mechanochemical reactions, quite realistically describes mechanochemical reactions under the action of EWP and, probably, mechanochemical reactions in general [4,11]. Indeed, if we assume that the elastic waves initiating the appearance of EMD are solitons (dislocation can be considered as a one-dimensional soliton), then the general scheme of a mechanochemical reaction looks similar to the following:

S → EMD → e^−^_K_, (*hω*)*_p_*, M^+^, M* → stable products

where S is a soliton, EMD is an electromagnetic–mechanical dipole, e^−^_K_ is polaron (an electron bound to phonons), (*hω*)*_p_* is a polariton, M^+^ is an ion and M* is an excited molecule. Such a scheme [4,11] agrees with and complements the mechanisms of phonon [12] and exciton dislocation [13] and the destruction of solids and ionic crystals, and also agrees with the ideas of the connection between the front of solid-phase reactions with internal mechanical stresses [14] and nonlinear waves [15]. All these assumptions led to the further logical development of research in the field of pulsed mechanochemistry.

A polymer matrix (polyethylene, polystyrene, ethylene copolymer with vinyl acetate) doped with 3,6-di-tert-butyl-quinone and 3,6-di-tert-butyl-pyrocatechin and chromium acetylacetonate (or chromium carbonyl) was used as an example of systems [43] in which there is possibility of obtaining coordination polymers when chromium chelate complexes are attached to polymer chains in mechanochemical processes occurring under the action of elastic wave pulses. It is shown that the elastic properties of polymer matrices affect the mechanochemical yields of chromium chelate complexes. A coordination polymer containing two-core complexes of niobium or manganese [6,44] with organic ligands catechol/ortho-quinone was obtained using a similar mechanochemical synthesis. The experimental EPR spectra of the manganese complex were analyzed by modeling using the quantum relaxation equation. This analysis revealed a triplet (full electron spin S = 1) ground state of a complex with two nonequivalent manganese ions bound to each other by spin exchange and magnetic dipole–dipole interaction. Using the density functional theory, the possible structures of the complex were calculated. The most probable structure is two paramagnetic manganese ions with different charge states bound via an oxygen atom of the Mn^2+^–O–Mn^4+^ type, surrounded by diamagnetic semiquinone ligands. Further study of such processes in the polymer matrix with the emergence of such complexes, as well as the study of their properties, required the improvement of the experimental base and the development of methods for analyzing fast mechanochemical processes.

## 3. Advanced Technique of Pulsed Mechanochemical Action

**Figure 3:**
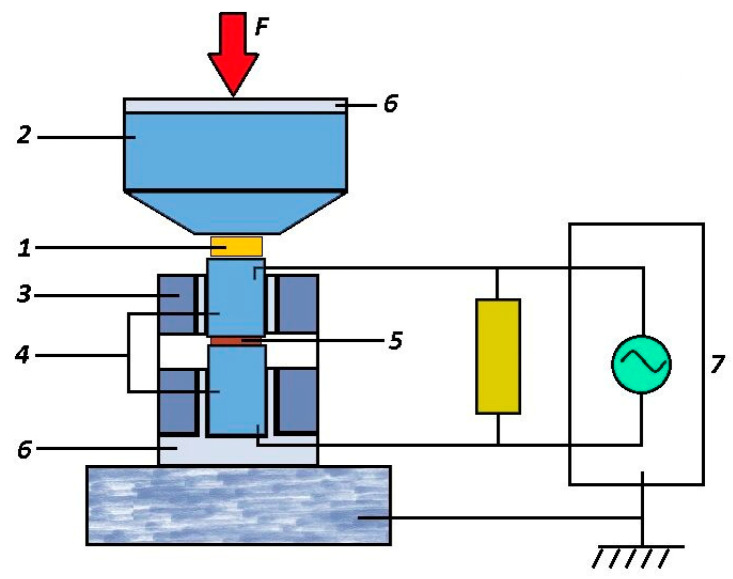
Diagram of an improved high-pressure cell (From [48] with permission).

To study fast mechanochemical processes, a high-pressure cell was improved upon (the scheme is given in Figure 3).

The cell (Figure 3) is isolated from the press equipment and consists of a Bridgman anvil (2 in Figure 3), a steel cage (3 in Figure 3) and punches (4 in Figure 3) isolated from the cage, between which the test sample is located (5 in Figure 3). In Figure 3, the isolation is indicated as 6. The punches are connected through a load resistance of 50 ohms to a Tektronix MSO 200 two-channel digital oscilloscope (7 in Figure 3). A sample of a standard polymer placed between the Bridgman anvil and the upper punch (1 in Figure 3) implements a RE to supply an external pulse of an elastic wave to the sample (5 in Figure 3).

The improvement of the research methodology made it possible to record the electromagnetic response during rapid physico-chemical processes in three different exposure modes:

1. In the RE mode, when only a sample (5 in Figure 3) is used, clamped between punches (4 in Figure 3) and realizing a RE;
2. In parametric mode, when an external elastic wave pulse arises from a sample of a standard polymer placed between the Bridgman anvil and the upper punch (1 in Figure 3); the test sample is located between the punches (4 in Figure 3) and does not realize a RE due to its low thickness;
3. In the RE mode due to a rapid pressure release (the discharge pulse is applied to the sample), when only a thin sample is used (5 in Figure 3) between the punches (4 in Figure 3), and the fixed pressure and its abrupt discharge are set by the design of the press equipment.

The cell (Figure 3) allows for the recording of the current pulse *J*(*t*) generated by the electrical component *E*(*t*) of electromagnetic radiation arising in the sample. Accordingly, a voltage pulse *U*(*t*) = *RJ*(*t*) is recorded by the oscilloscope. In this case, it is possible to determine the power (intensity) of electromagnetic radiation, since *J*(*t*)~*E*(*t*), and the power (intensity) of electromagnetic radiation *I*(*t*)~[*E*(*t*)]^2^; thus, *I*(*t*)~[*J*(*t*)]^2^. In addition, if necessary, the piezoelectric sensor can record the pressure drop process. To do this, the piezoelectric sensor is placed under the lower punch.

The experiments described below were carried out using this cell. It should be noted that the concentrations of fillers in the polymer matrix were used after preliminary experiments, which made it possible to establish concentration intervals for the implementation of the SR process at the same pressure *P_RE_* = (2.0 ± 0.15) GPa.

## 4. Radio-Frequency Superradiance Effect in the Rheological Explosion Mode

In [49,50,51], it was shown for the first time that two phenomena, RE and SI, can occur simultaneously. Composites based on industrial polystyrene PS with molecular weight *M_W_*~89,250 were studied. Composite samples (*PS* + Co(acac)_2_, *PS* + Mn(acac)_2_) contained complexes of [Co(acac)_2_] and [Mn(acac)_2_] in the following quantities (*N* complexes in 1 cm^3^ composite): 15·10^20^; 0.3·10^20^; 0.6·10^20^; 0.8·10^20^; 1.0·10^20^; 1.2·10^20^. Composite samples (*PS* + QH_2_) contained a number of 2QH_2_ complexes, from which radical pairs are formed (*N* complexes in 1 cm^3^ of the composite): 0.15·10^20^; 0.3·10^20^; 0.6·10^20^; 0.8·10^20^; 1.0·10^20^; 1.2·10^20^. The amount of QH_2_ was enough for the reaction M(acac)_2_ + 2QH_2_ → M(QH)_2_ + 2H(acac) (M = Co, Mn) to yield 100%.

All samples were tablets with a diameter of 10 mm and a thickness of 2 mm, after pressure-molding the initial mixture at 190 °C, and the above concentrations of fillers were taken so that the rheological explosion occurred at the same pressure *P_RE_* = (2.0 ± 0.15) GPa.

**Figure 4:**
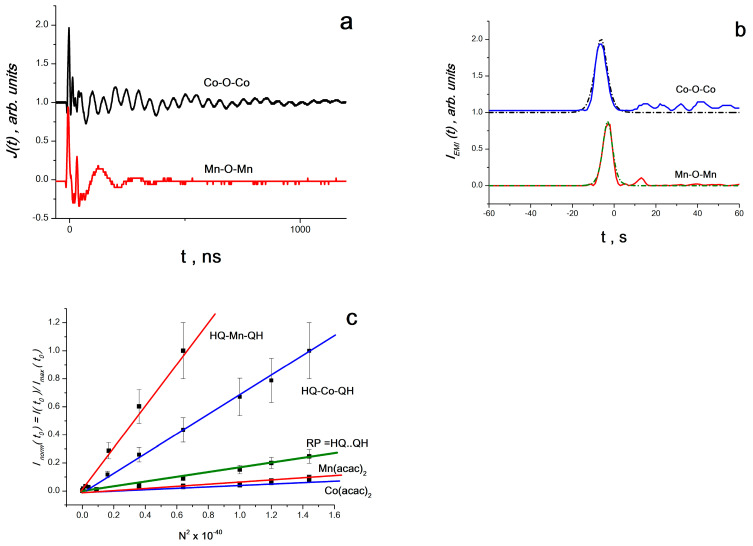
(**a**) normalized time sweep of the electrical signal; (**b**) normalized time sweep of the generated power at the load resistance; (**c**) normalized dependence of the ratio *I_EMR,norm_* = *I_EMR_*(*t*_0_)/*I_EMR,max_*(*t*_0_) on *N*^2^ (*N_MAX_* = 1.2·10^20^—the maximum number of complexes in 1 cm^3^) on the square of the concentration of complexes in 1 cm^3^ of the composite (From [51] with permission).

During the rheological explosion, an alternating current *J*(*t*) was registered in the samples (Figure 4a) generated by the electrical component *E*(*t*) of electromagnetic radiation from the products of mechanochemical reactions. Since *J*(*t*)~*E*(*t*), and the power (intensity) of electromagnetic radiation *I*(*t*)~[*E*(*t*)]^2^, then *I*(*t*)~[*J*(*t*)]^2^. Figure 4b shows the characteristic peaks of the intensity of electromagnetic radiation *I_EMR_*~[*J*(*t*)]^2^ for composites *PS* + Co(acac)_2_ + 2QH_2_ and *PS* + Mn(acac)_2_ + 2QH_2_ for *N* = 0.8·10^20^ for Co(acac)_2_ or Mn(acac)_2_ in 1 cm^3^ composite. The dotted line shows the anamorphoses of the maximum bands *I_EMR_*, for which the shape of the line was calculated according to Equation (7). Here, *I_EMR_* = *I_EMR_*(*t*) is the intensity of electromagnetic radiation at time *t*; *I*_0,*EMR*_ = *I*_0,*EMR*_(*t*_0_) is the maximum intensity of electromagnetic radiation at time *t*_0_.

It was found that the intensity of the maximum peaks of *I_EMR_*(*t*_0_) (at *t* = *t*_0_) is proportional to the square of the concentration of *N*^2^ complexes (*N* varied from 0.6·10^20^ to 1.2·10^20^ complexes in 1 cm^3^). This is clearly seen from Figure 4, which shows the dependence of the normalized amplitude, *I_EMR,norm_* = *I_EMR_*(*t*_0_)/*I_EMR,max_*(*t*_0_), on *N*^2^ (*N_MAX_* = 1.2·10^20^—the maximum number of complexes in 1 cm^3^).

For the range of the above concentrations of complexes, it was found that (1) the shape of the radiation band line corresponds to Equation (7), characteristic of superradiation processes—an exponential symmetric rise and fall [16,17,18]; (2) the radiation intensity is proportional to the square of the concentration of complexes—a similar quadratic dependence is also characteristic of superradiation processes [16,17,18]; (3) the process takes place in the frequency range of 60–200 MHz, in which any molecular system interacts with all other radiating systems through a common electromagnetic field and forms a single system [16,17,18]. It can be assumed that the observed process is due to spontaneous radio frequency generation of chemical reaction products and corresponds to SR in the radio frequency range.

It was suggested in [49,50,51] that triplet–triplet annihilation is responsible for the observed SR processes during RE. Therefore, ab initio calculations of the electronic and spin structure of the following complexes were carried out: Co(acac)_2_, Mn(acac)_2_, HQ...QH and HQCo–O–CoQH, HQMn–O–MnQH. Calculations were carried out using the Becke–Lee–Yang–Parr density functional DFT (B3LYP) method with the Kohn–Sham equation and the basic set of two-exponential Dunning–Hay atomic functions for heavy elements (LanL2DZ), according to the GAUSSIAN98 program [51]. Calculations have shown that all these complexes can exist in both singlet and triplet states, and the singlet–triplet transition is realized with minor structural transformations. Complexes Co(acac)_2_ and Mn(acac)_2_ are Jahn–Teller systems that, under the influence of elastic wave pulses (EWP) from a RE, experience structural changes with further transformation into acetylacetone and cobalt ion. In the composite *PS* + Co(acac)_2_ + QH_2_, cobalt ions and HQ radicals form particles that can enter into triplet–triplet annihilation reactions through an oxygen atom with the formation of cobalt oxide compounds and spatially hindered phenols or with the formation of HQCo–O–CoQH and HQMn–O–MnQH complexes. Such two-spin objects, according to ideas that have been intensively developed recently, should have a magnetoelectric effect, and realize (at least for the duration of their existence) magnetic ordering with electric and magnetic polarization of the volume by elastic waves [49,50,51]. It is the presence of these properties in complexes that leads to the fact that the SR effect in molecular structures such as HQCo–O–CoQH and HQMn–O–MnQH is much more intense [51] than in radical pairs or Jahn–Teller complexes of Co(acas)_2_ or Mn(acas)_2_, which is clearly seen from Figure 4c.

The above data allow us to conclude that during a RE, the electron spin Zeeman reservoir is inversely populated, becoming the source of the observed electromagnetic SR that occurs due to the annihilation of triplet excitations. The intensity of the SR process is directly related to the electronic properties and structure of the two-spin intermediates [52,53,54,55,56,57,58].

## 5. Radio-Frequency Superradiance Effect in Parametric Mode from an External Pulse of an Elastic Wave

Since the SR effect involving molecular structures such as HQCo–O–CoQH or HQMn–O–MnQH is much more intense than those in radical pairs (HQ...QH) or Jahn–Teller complexes Co(acas)_2_ or Mn(acas)_2_ [49,50,51], composites (*PS* + Co(QH) were further investigated in detail (*PS* + Co(QH)_2_ and *PS* + Mn(QH)_2_) [48,59,60,61]. The studies were carried out in a parametric mode, i.e., from an external pulse of an elastic wave, which arises from a standard polymer sample placed between the Bridgman anvils and the upper punch (1 in Figure 3) at the same pressure *P_RE_* = (2.0 ± 0.15) GPa, while between the punches (4 in Figure 3), a test sample is placed that does not realize a RE due to its low thickness.

The synthesis of Co(QH)_2_ and Mn(QH)_2_ was carried out according to the well-known method [59], where QH is a ligand based on QH_2_–3,6-di-tert-butylpyrocatechin. Composites were obtained by mixing Co(QH)_2_ or Mn(QH)_2_ with a polystyrene matrix (PS). Concentrations of binuclear complexes of cobalt or manganese in 1 cm^3^ were equal to 0.1·10^20^; 0.2·10^20^; 0.3·10^20^; 0.4·10^20^; 0.5·10^20^; 0.6·10^20^; and 0.7·10^20^.

**Figure 5:**
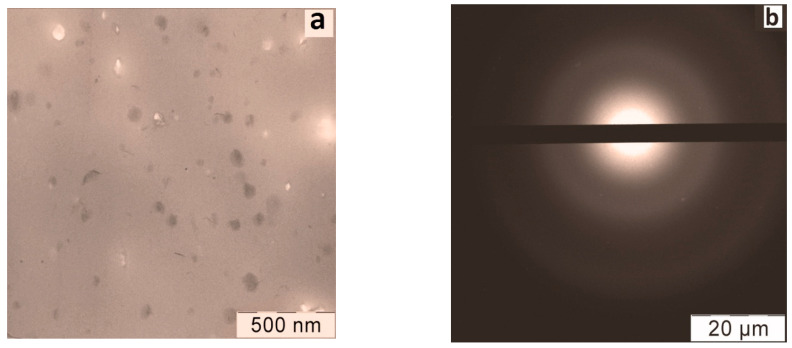

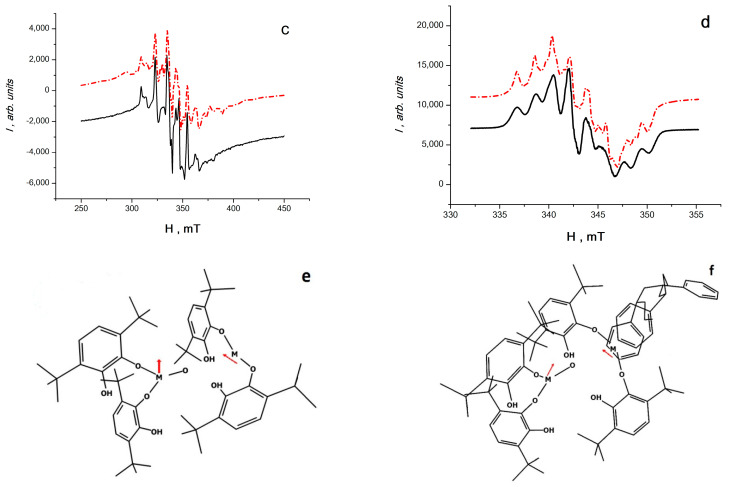
Microphotographs of the nanocomposite—BC Mn complexes in polystyrene matrix, obtained by transmission electron microscopy in the light field mode (**a**) and electron diffraction (**b**). ESR spectra of BC Mn (**c**) and BC Co (**d**) complexes in polystyrene matrix, at 77K (-----) and their theoretical anamorphoses (----). Geometric structure of the individual complex (HQ)_2_M··O=M(QH)_2_ (**e**) and stabilized in polystyrene matrix (**f**) after full optimization by DFT (M = Co, Mn) (From [48] with permission).

It was found that the cobalt (or manganese) compounds were distributed in the polymer matrix in the form of 2D nano-objects—plates with transverse dimensions of about 50–100 nm and a thickness of ~1–2 nm (Figure 5a). The composites did not have a crystalline structure, which is clearly seen from the electron diffraction pattern (Figure 5).

The ESR spectra of stable radical products in the initial systems (*PS* + Co(QH)_2_ or *PS* + Mn(QH)_2_) are shown in Figure 5c,d (solid lines). Anamorphoses of these multicomponent ESR spectra were constructed according to a method described previously [6], where the theoretical ESR spectra are calculated as the result of a multiparametric solution of a quantum relaxation equation with Hamiltonian (13):

(16) $$H=g_{a}\beta HS^{a}+g_{b}\beta HS^{b}+JS^{a}S^{b}+G\left[S^{a}S^{b}\right]+D\left[S^{a}S^{b}-3S_{z}^{a}S_{z}^{b}\right]+E\left(S_{x}^{a}S_{x}^{b}-S_{y}^{a}S_{y}^{b}\right)+A_{a,iso}I^{a}S^{a}+A_{b,iso}I^{b}S^{b}$$

where *g_a_* *=* (*g_ax_* + *g_ay_* + *g_az_*)/3, *g_b_* = (*g_bx_* + *g_by_* + *g_bz_*)/3, *A_a,iso_* = (*A_ax_* + *A_ay_* + *A_az_*)/3, *A_b,iso_* = (*A_bx_* + *A_by_* + *A_bz_*)/3, *J* and *G* are constants of isotropic and anisotropic exchange interaction, and *D* and *E* are constants of dipole–dipole and spin–spin interactions.

Computer analysis has demonstrated that the theoretical spectrum can only be calculated if the object being studied by the ESR method consists of two unequal paramagnetic centers *a* and *b* connected by exchange interaction, or in other words, if the object is composed of these two centers, i.e., a complex made up of two paramagnetic centers known as a binuclear cluster (BC). The calculations give *g*-factors, hyperfine interaction constants, and the widths of the associated ESR lines. From the assumption that the values of the isotropic constants are directly proportional to the spin densities (SD) *σ_a_* and *σ_b_*, it follows that on the first cobalt (manganese) core, the fraction of SD is 0.69 (0.75), and on the second, it is 1.31 (1.25). Thus, the first center for both metals has a stronger bond with polymer chains, since its spin density *A_a,iso_* is less than that at the second center *A_b,iso_*, and Δ*H_ax_* is greater than Δ*H_bx_* at the second center (Δ*H_y_* and Δ*H_z_* are approximately equal for both centers).

The splitting parameters in zero field for BC Co (BC Mn) are equal to *B* = 4.90 (19.48) mT and *E* = 5.83 (6.21) mT, the constants of the scalar exchange interaction are equal to *J* = 10.15 (24.20) mT, and the anisotropic exchange interaction *G* = 43.98 (23.06) mT. If the constants of scalar and anisotropic exchange interactions exceed the value of dipole constants, then this indicates the indirect nature of spin exchange [61,62,63,64,65,66,67], i.e., that cobalt and manganese ions are interconnected, for example, through an oxygen anion. Thus, it is most likely that the ESR spectra can be correlated with complexes of the BC Co (or BC Mn) type, i.e., with complexes Co(QH)**_2_**–O–Co(QH)**_2_** and Mn(QH)**_2_**–O–Mn(QH)_2_.

Quantum chemical calculations of molecular structures were also carried out—ab initio calculations of the electron and spin structure of complexes Co(QH)_2_–O–Co(QH)_2_ and Mn(QH)_2_–O–Mn(QH)_2_ (Figure 5e) and complexes where one of the metal atoms is embedded in a polymer chain between two benzene rings (Figure 1f). Calculations were carried out by the Becke–Lee–Yang–Parr density functional DFT (B3LYP) method using the Kohn–Sham equation and the basic set of two-exponential Dunning–Hay atomic functions for heavy elements (LanL2DZ), according to the Gaussian 09 program [68]. Calculations have shown that these complexes can exist in both singlet and triplet states and that the singlet–triplet transition is realized with insignificant structural conformations, for example, when an oxygen atom shifts between cobalt (or manganese) atoms. Similar two-spin objects have a magnetoelectric effect [41,42].

**Figure 6:**
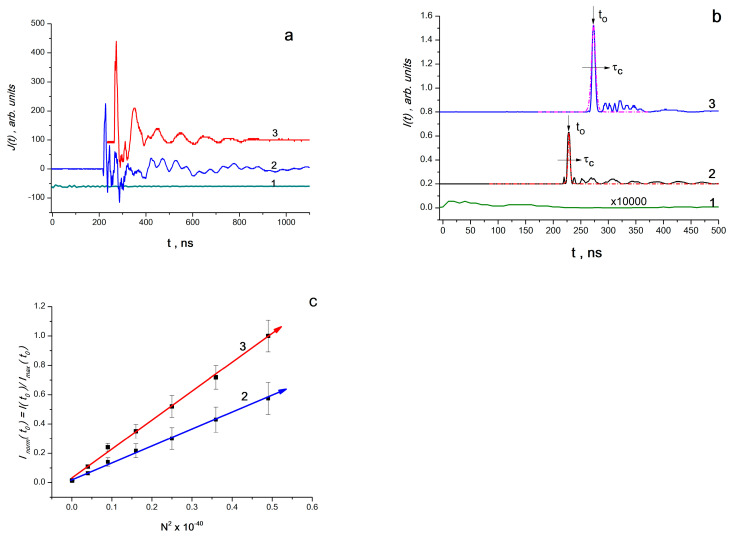
(**a**) time scan of the electrical signal *J*(*t*)~*E*(*t*) for PS matrix (1) and samples containing BC Co (2) and BC Mn (3); (**b**) time scan of the normalized signals *I*(*t*)~[*E*(*t*)]^2^ for PS matrix (1) and samples containing BC Co (2) and BC Mn (3); (**c**) is the normalized dependence of *I*(*t*_0_) on the square of the concentration of complexes in 1 cm^3^ of the composite (From [48] with permission).

It was found that when an elastic wave pulse (EWP) is applied to the sample, an alternating current *J*(*t*) is recorded (Figure 6a), generated by the electrical component *E*(*t*) of electromagnetic radiation. Since *J*(*t*)~*E*(*t*), the power (intensity) of electromagnetic radiation *I*(*t*)~[*E*(*t*)]^2^; thus, *I*(*t*)~[*J*(*t*)]^2^ (Figure 6b).

It was found that the intensity of peaks *I*(*t*_0_) (at *t = t*_0_) is proportional to the square of the concentration of BC complexes *N*^2^ (*N* varied from 0.1·10^20^ to 0.7·10^20^ for binuclear complexes (BC) in 1 cm^3^). This is clearly seen from Figure 6c, which shows the dependence of the normalized amplitude *I*(*t*_0_) on *N*^2^ for BC of cobalt and manganese (as *N_MAX_*, the maximum value for the BC of manganese is taken). Knowing the initial concentration of complexes and determining the pulse duration of SK from the dependence *I* = *I*(*t*) (for example, at *N* = 0.6·10^20^ BC in 1 cm^3^ for BC Co and BC Mn *τ_c_*, pulse duration is 4 and 5 ns, respectively), it is possible to determine pulse delay time *t*_0_ = *τ_c_lnN*, which equals, for BC Co and BC Mn, 227 and 273 ns, respectively (Figure 6b).

Accordingly, for the concentration range 0.1·10^20^–0.7·10^20^ of BC in 1 cm^3^, it was found that: (1) the shape of the radiation bands corresponds to Equation (7), characteristic of superradiation processes—an exponential symmetric rise and fall; (2) the intensity of radiation is proportional to the square of the concentration of complexes—a similar quadratic dependence is also characteristic of superradiation processes (Equation (7)).

**Figure 7:**
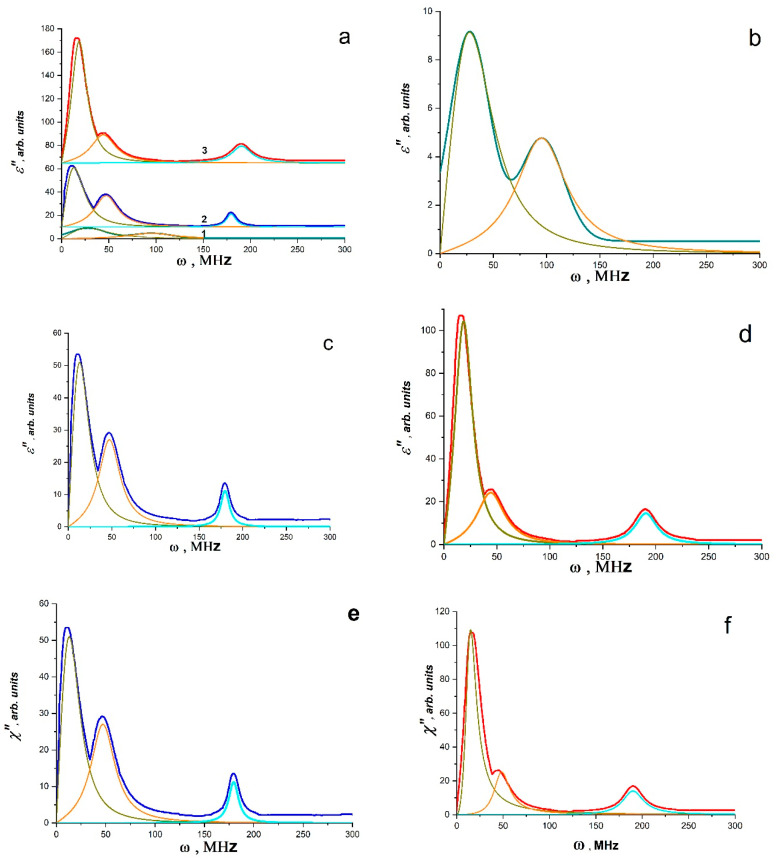
(**a**) Fourier images of electrical signals of PS matrix (1), PS–BC Co (2) and PS–BC Mn (3); absorption bands obtained for PS matrix (**b**), PS–BC Co (**c**) and PS–BC Mn (**d**) by the Lorentz method and absorption bands for PS–BC Co (**e**) and PS–BC Mn (**f**) obtained by the LLB method (From [48] with permission).

Fourier analysis of pulses *J*(*t*)~*E*(*t*) showed that the observed processes of radio-frequency SR lie in the wavelength range from 0 to 200 MHz, and radio-frequency radiation (not SR!) from the control samples of the matrix polymer (polystyrene) lies in the range from 0 to 100 MHz (Figure 7a,b). At the same time, the intensity of the RF radiation of the matrix polymer is less than the intensity of SK in composites with BC Co or BC Mn by four to five orders of magnitude (Figure 6b).

As shown in Figure 7a–f, band spectra are depicted in the Fourier images of signals *E~E*(*t*)~*J*(*t*). Individually excited molecules that are weakly or not at all bound to one another produce band spectra (molecular gas). Atomic electronic transitions as well as the vibrational movements of the atoms within molecules both contribute to the radiation’s occurrence. Therefore, it is conceivable that upon exposure to EWP, local defrosting of the molecular mobility of macromolecule fragments occurs (the effective local temperature is much higher than the glass transition temperature), with possible partial ionization of individual atoms and the formation of regions having the properties of molecular gas and partly electron or ion plasma. These regions ought to produce electromagnetic radiation at frequencies corresponding to the natural fluctuations of charges in this volume. It appears that the formation of regions with various sizes and oscillation frequencies, such as those in the polymer volume, at the interface, etc., is what causes the presence of different bands in the radiation spectrum. Typically, a harmonic oscillator model with attenuation is used to describe such spectra. It is clear that polarization fluctuations are responsible for the high-frequency oscillations *E*(*t*), as they are connected to fluctuations of both free charges and charges bound to the composite’s molecular components. Thus, the data for PS and PS - BC Co or PS - BC Mn systems can be interpreted as the spectra of the dielectric losses of the system at the moment of the RE and can be analyzed by the methods of dielectric spectroscopy operating in terms of electric dipoles, i.e., using Equations (11) and (12). Since the polarization changes are caused by a system of dipoles based on an ensemble of non-collinear spins (a system of Dzyaloshinskii–Moriya dimers, which are BC Co and BC Mn), it is possible to analyze the spectra for *PS* - BC Co or *PS* * BC Mn systems using the LLB equation (Equations (13)–(15). Figure 7a–f presents the results of the approximation of the spectra using the above equation for *ε*″(*ω*) (Figure 7a–d) and for *χ*″(*ω*) (Figure 7e,f) according to Equations (13) and (15). The data obtained are shown in Table 1.

The parameter *k* = 2*α*/*ω_R_* plays an important role in establishing the relaxation mechanism. For systems analyzed using the LLB equations, *k* = 2*ω_r_*/*ω_R_* or *k* = 2*ω_H_*/*ω_R_*.

When 0 < *k* < 1, there is only resonant absorption (or emission) in the system. When *k* > 1, there is a significant contribution of relaxation processes, which increases with increasing *k*, while the peak also widens. As can be seen from the values of *k* in Table 1, for all low-frequency bands (the maximum frequency is ~10 MHz), the contribution of relaxation processes is significant, i.e., polar groups and segments of molecules are involved in absorption. For all bands in the range of 20–210 MHz (Table 1), only resonant absorption (or emission) takes place. For systems containing BC Co and BC Mn, it can be assumed that all peaks are associated with superradiation processes, since their intensity is significantly higher than the intensity of the peaks of the polymer matrix.

**Figure 8:**
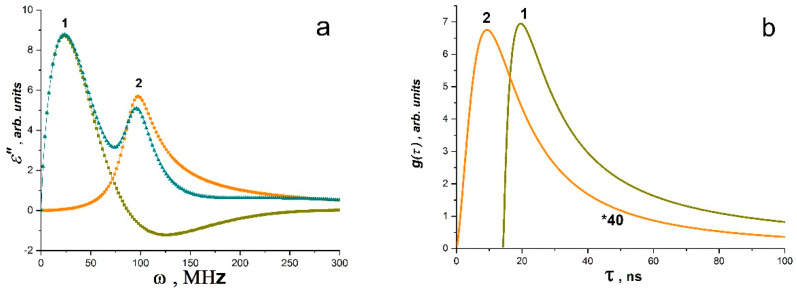

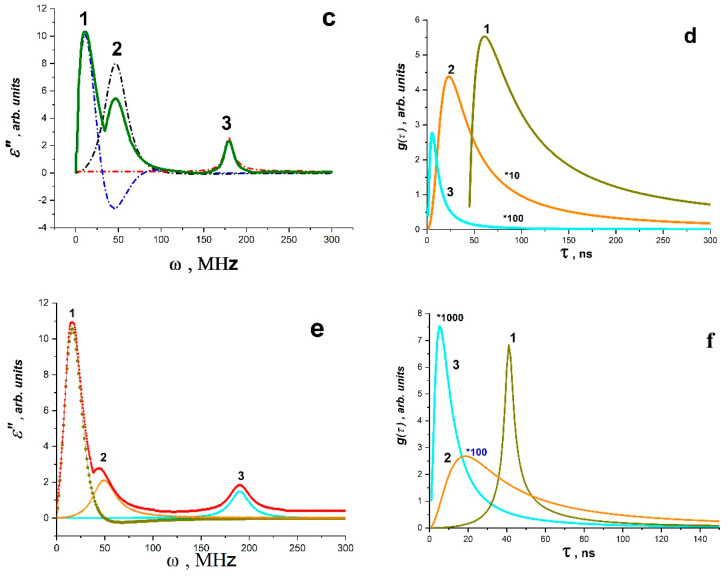
Dependence of permittivity *ε*″ on frequency according to the Havriliak–Negami model for PS matrix (**a**), PS–BC Co (**c**), PS–BC Mn (**e**). Curves of the relaxation time distribution *g*(*τ*) for PS matrix (**b**), PS–BC Co (**d**), PS–BC Mn (**f**). The numbers of absorption bands and curves *g*(*τ*) correspond to the numbers in brackets in Table 2, (**f**) curves 2 and 3 *y*-axes multiplied by 100 and 1000 respectively (From [48] with permission).

However, the analysis based on the polarization and LLB equations does not allow for evaluation of the relaxation properties of these systems. The Havriliak–Negami method allows for the analysis of these characteristics [69]. From the analysis by the Havriliak–Negami method, it can be seen that the bands of radio-frequency radiation lines have different relaxation characteristics (Equations (17) and (18)). Moreover, the relaxation characteristics differ for different sections of each line. This is clearly seen from Figure 8a–f. Figure 8a,c,e shows curves approximating the lines of radio-frequency radiation according to Equation (17) and (18). The approximation process allows us to obtain the initial data (they are shown in Table 2), which, according to Equation (14), make it is possible to construct the curves of the relaxation time distribution as a function of time. It can be seen that the relaxation time distributions are quite narrow and have a long tail on the high-frequency side. In Table 2, the characteristic values of the relaxation time in the radiation band maxima are listed. It is evident that the relaxation times of bands with maxima at 180 MHz (BC Co) and 189.7 MHz (BC Mn) are 4–12 times slower than those of bands with maxima in the range 0–100 MHz and associated with polarization processes affecting the polymer matrix. It should be noted that the time it takes for bands in the 0–100 MHz range to relax increases for composites compared to the time it takes for bands in the pure polymer matrix and the intensity of these radiation bands in the 0–100 MHz range is noticeably higher than the intensity of the radiation bands of the pure matrix. Since one of the metal atoms is stabilized by benzene rings of the polystyrene matrix, similar to the ferrocene structure, bands with a maximum in the range of 0–100 MHz likely experience an amplification (trigger effect) because of the interaction of BC Co and BC Mn with polymer chains (Figure 5f). The characteristic relaxation times for the bands of 180 (189.7) MHz (for BC Co and BC Mn) are equal to 4–5 ns and coincide with the characteristic times of *t_c_* (pulse durations of SR for BC Co and BC Mn). This indicates that the polymer matrix is actively involved in the SR process. It is likely that polymer chain vibrations are coherent as well, and the polarization processes that take place in this situation cause the phonon–polariton mechanism, which was predicted in [4,11], to pump the spin reservoir of the binuclear clusters BC Co and BC Mn.

(17) ε″=Δε[1+iff0a]b

(18) $$g\left(\tau \right)=\frac{1}{\pi }\frac{{\left(\tau /\tau_{H}\right)}^{\alpha \gamma }\sin\left(\gamma \theta \right)}{{\left[{\left(\tau /\tau_{H}\right)}^{2\alpha }+2{\left(\tau /\tau_{H}\right)}^{\alpha }\cos\left(\alpha \pi \right)+1\right]}^{\gamma /2}}$$

where $\theta =arctan\left|\frac{\sin\left(\gamma \pi \right)}{{\left(\tau /\tau_{H}\right)}^{\alpha }+\cos\left(\alpha \pi \right)}\right|$.

## 6. Radio-Frequency Superradiance Effect in the Mode of Rapid Pressure Release

After two–spin systems were investigated [48,49,50,51,60], the question arose whether it was possible to implement SR under pulsed mechanical activation in multi-spin systems, for example, in a four-spin system, which is a molecular magnet. Such interest is associated with the assumption that in a multi-spin system, the process of SR during mechanical activation is likely to have a higher intensity due to spin catalysis.

**Figure 9:**
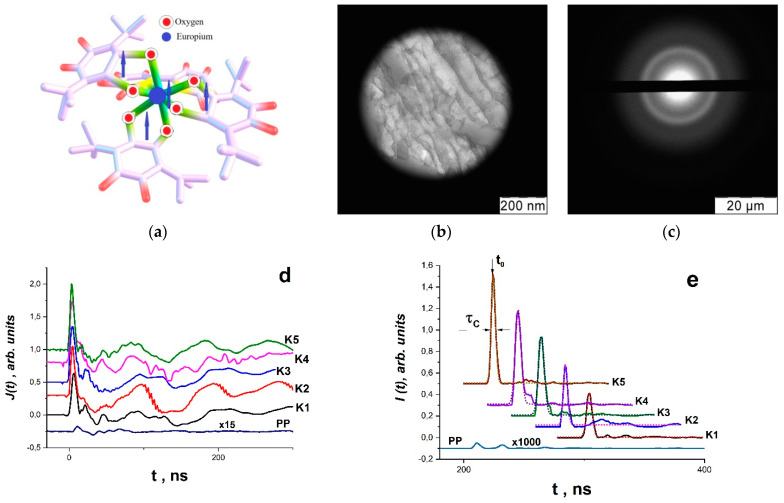

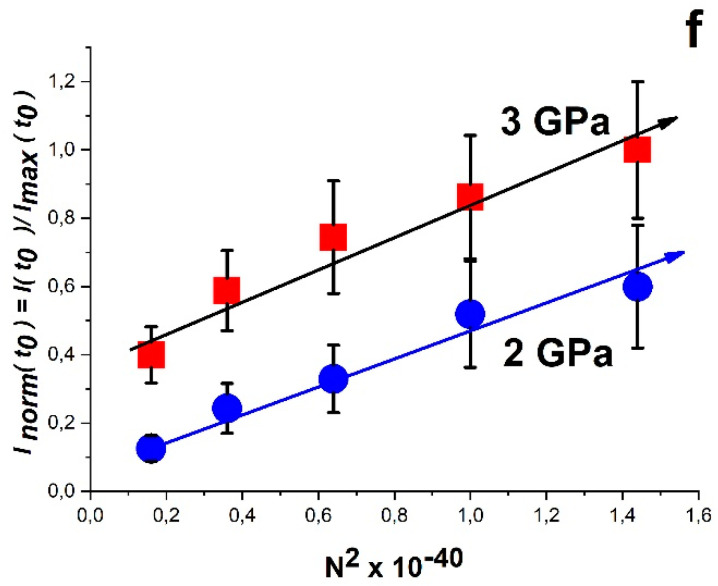
(**a**) structure of Eu(III)(SQ)_3_·bipy complex; (**b**) microphotograph and (**c**) electronogram of composite sample K5 (*PS*–Eu(III)(SQ)_3_·bipy) before mechanical activation; (**d**) time scan of normalized signals *J*(*t*) for composites K1, K2, K3, K4 and K5 and *PS*; (**e**) time sweep of normalized signals *I*(*t*)~[*E*(*t*)]^2^ for composites K1, K2, K3, K4 and K5 and *PS*; (**f**) normalized dependence *I*(*t*_0_) on the square of the concentration of Eu(III)(SQ)_3_·bipy complexes for composites K1, K2, K3, K4 and K5. Curves were normalized to the maximum value *I*(*t*_0_) for K5 (From [48] with permission).

As a result, composites of Eu(III)(SQ)_3_·bipy complexes in polystyrene (specifically, composites K1, K2, K3, K4 and K5) were created, with concentrations of 0.4 × 10^20^, 0.6 × 10^20^, 0.8 × 10^20^, 1.0 × 10^20^ and 1.2 × 10^20^ cm^−3^. It is described in [70] how to synthesize this complex and how it is structured. A molecular magnet with four unpaired electrons, one of which is on the Eu(III) ion and the other three of which are on SQ ligands, was also demonstrated in [70] (Figure 9a).

For 10 min, Eu(III)(SQ)_3_·bipy and PS were blended in the Pulverisette 0 ball vibrating micro-mill (Germany). At 190 °C, the resulting mixture was pressure-molded. Under uniaxial compression at 5 GPa, samples with a diameter of 12 mm and a thickness of 1 mm exhibited no RE. Before mechanical activation, Figure 9b,c displays the micrograph and electronogram of the K5 (PS–Eu(III)(SQ)_3_·bipy) composite sample. To study SR pulses under mechanical action, a special high-pressure cell was employed (Figure 3). REU, or rheological unloading explosion, was the impact mode [71,72] due to the rapid pressure drop.

It was found that radio-frequency superradiance (RSR) can be implemented for all samples K1–K5 under REU conditions at 2 and 3 GPa [71,72]. At the same time, an alternating current *J*(*t*) was recorded (Figure 9d), generated by the electrical component *E*(*t*) of electromagnetic radiation. Figure 9e shows the pulses of electromagnetic radiation *I*(*t*)~[*E*(*t*)]^2^~[*J*(*t*)]^2^~[*U*(*t*)]^2^, reaching maximum intensity at *t* = *t*_0_. It can be seen that the shape of t radiation bands corresponds to the law, characteristic of SR processes—an exponential symmetric rise and fall (in Figure 9e, anamorphoses according to Equation (7) are given in dotted lines). It was found that the intensity of peaks *I*(*t*_0_) (at *t* = *t*_0_) is proportional to the square of the concentration of complexes. This is clearly seen from Figure 9f, which shows the dependence of the normalized amplitude *I_norm_*(*t*_0_) = *I*(*t*_0_)/*I_MAX_*(*t*_0_) on *N*^2^ for Eu(III)(SQ)_3_·bipy complexes. *I_MAX_*(*t*_0_) corresponds to the maximum value of the RSR pulse for the K5 composite sample at 3 GPa, where *I_MAX_*(*t*_0_) is for the sample containing 1.2 × 10^20^ Eu(III)(SQ)_3_·bipy complexes in 1 cm^3^ polystyrene, i.e., the maximum concentration of europium complexes. From Figure 9f, it can be seen that using the RSR mode showed that the RSR process can be implemented for all samples K1–K5 under REU conditions at 2 and 3 GPa (Figure 9f). The intensity of peaks *I*(*t*_0_) for samples K1–K5 is almost four orders of magnitude higher than the intensity of the pulse from pure polystyrene, wherein a rheological discharge explosion occurs at 3 GPa. The signals of the matrix polymer lack these distinctive peaks with the structure denoted by Equation (7) (Figure 9d,e). It was assumed that *τ_c_* is the pulse width at half-height and that 2*τ_c_* is the pulse duration when calculating *t*_0_ = 2*τ_c_lnN* using the experimental value of *τ_c_*. It should be noted that the proportion of paramagnetic complexes in the samples that were converted into non-paramagnetic particles is 70% of the total number of complexes in all samples and is linearly proportional to *N*, the total number of complexes in the composite during synthesis in terms of cm^−3^. It was discovered that as the concentration of complexes in the sample increases, the pulse delay time decreases; in our case, this is true for K1–K5, where the pulse delay times are 300, 280, 260, 240 and 200 ns, respectively (Figure 9e).

**Figure 10:**
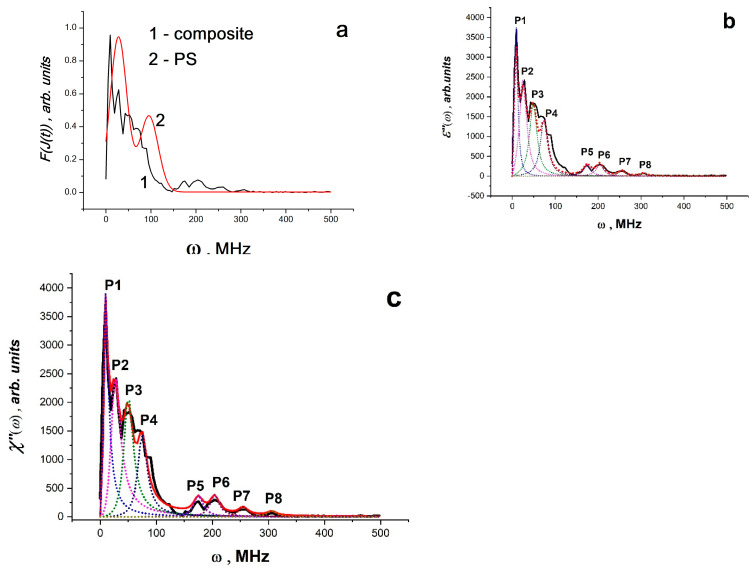
(**a**) normalized Fourier image of the electrical signal for the composite K5 (1) and PS (2). (**b**) Fourier image of the electrical signal for the composite K5 and the radiation bands obtained by the Lorentz method (their parameters are given in Table 1). (**c**) Fourier image of the electrical signal for the composite K5 and the bands the radiations obtained by the Landau–Bloch–Blombergen method (their parameters are given in Table 3) (From [48] with permission).

When the pulses *J*(*t*)~*E*(*t*) were subjected to Fourier analysis, it became clear that the observed processes of RSI emission occurred between 0 and 300 MHz, i.e., in the band of radio frequencies (Figure 10a). Figure 10a shows what is happening. Striped spectra are depicted in Fourier images of signals *EE*(*t*)*J*(*t*). The composite’s normalized Fourier spectrum of the matrix polymer polystyrene (spectrum 2) and the composite (spectrum 1) are vastly dissimilar in structure. In other words, it is possible to argue that the introduced Eu(III)(SQ)_3_·bipy complex is the cause of the difference. As is well-known, excited individual molecules that are weakly or not at all bound to one another emit striped spectra. Atomic electronic transitions and the vibrational movements of the atoms within molecules both contribute to the radiation’s occurrence. Therefore, it is conceivable that local thawing of the molecular mobility of macromolecule fragments, destabilization of paramagnetic complexes introduced into the polymer matrix, ionization processes and the formation of an ensemble of free electrons occur when exposed to IUV. The inherent fluctuations of charges in this situation cause electromagnetic radiation to occur at certain frequencies. It is clear that polarization fluctuations are responsible for the high-frequency oscillations *E*(*t*), as they are linked to fluctuations of charges both free and bound to the composite’s molecular components. It is obvious that their electric dipole moment **D** is associated with their spin system, i.e., **D** ∝ **R** × (**S***a* × **S***b*), where **R** is the relative position of two arbitrarily taken spins in the complex (Eu(III)(SQ)_3_·bipy). Since **R** changes in time, the vectors of dipole moments **D** and the polarization vectors **P** and, accordingly, the magnetic moments of the system **M**, which are associated with the spin ensemble, change accordingly [25]. Using Equations (11–12) and (13–15), the Fourier spectrum for K5 at 3 GPa for each radiation band used to create the total spectrum was calculated. The results are displayed in Figure 10b,c. Table 1 contains the necessary initial data for the calculations.

**Figure 11:**
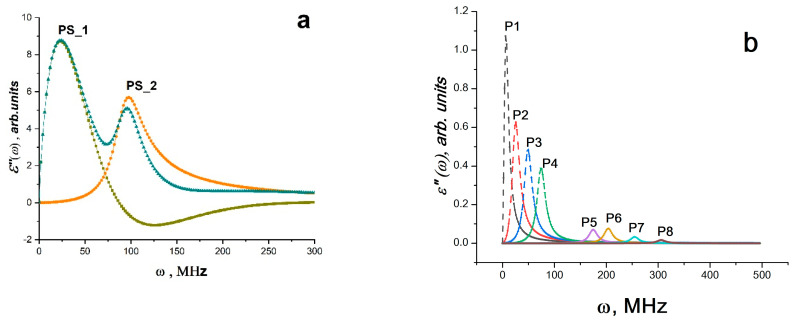

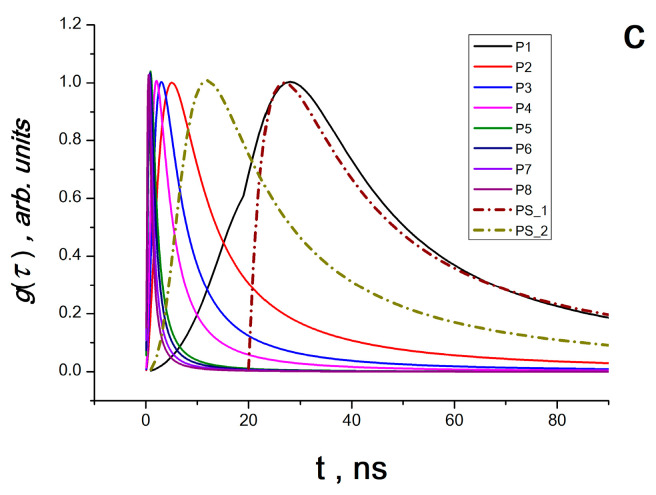
(**a**) Fourier image of the electrical signal for PS and the radiation bands obtained by the Havriliak–Negami method (the parameters are given in Table 4). (**b**) Fourier approximation of the image of the electrical signal for the composite K5 according to the Havriliak–Negami model (the band parameters are given in Table 4). (**c**) Relaxation time distribution curves *g*(*τ*) for PS matrix and composite K5. The numbers of the radiation bands and curves *g*(*τ*) correspond to the numbers in Table 4 (From [48] with permission).

However, the analysis of signals from the RE of K1–K5 composites and matrix polymer based on Equations (11)–(12) and (13)–(15) does not allow for the evaluation of the relaxation properties of the systems, while the Havriliak–Negami model can do this [60]. Since the spectrum description formalism within the framework of the Havriliak–Negami method works with a single spectral line regardless of whether this line belongs to the radiation or absorption process, the radio-frequency SR bands can be analyzed using the Havriliak–Negami method according to Equations (13) and (14) [60]. The Fourier approximation of the image of the electrical signal for the composite K5 and for the polystyrene matrix according to the Havriliak–Negami model is shown in Figure 11a,b. The data are shown in Table 4.

The relaxation time distribution curves *g*(*τ*) for the composite bands (in our case, K5) and for the polystyrene matrix can be built using the coefficients *log* ω_0_, Δ*ε*, *a* and *b* from Table 4. These curves are shown in Figure 11c, and are used to calculate the relaxation times in the radiation band maxima *τ_0_* (displayed in Table 4). On the side of high frequencies, the relaxation time distributions can be seen to have a long tail. The relaxation curves for polystyrene (curve PS_1) and for composite (curve P1) also coincide, as shown in Figure 11c. It also shows that the vibrations of the polystyrene matrix activate the polarization vibrations of the europium complex, demonstrating the direct involvement of the polymer matrix in the processes.

## 7. The Possible Mechanism of Radio-Frequency Superradiance Effect

**Figure 12:**
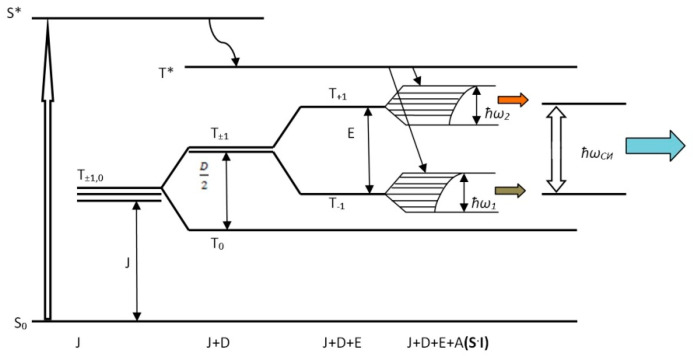
Energy diagram of population inversion of levels T_+1_ and T_−1_ (the length of horizontal segments is proportional to the population of levels when splitting T_+1_ and T_−1_ by the mechanism of spin–spin interaction) (From [48] with permission).

As was previously demonstrated, it is possible to use techniques of dielectric spectroscopy, the Lorentz equation and the LLB equation for weak ferromagnets to analyze the frequency and relaxation characteristics of the SR process. Since SR bands correspond to the ESR spectra parameters, which lie in the range of 0.01–7.0 mT, between 3 and 210 MHz, the data in this case support the assumptions made in [71,72,73] that the spin reservoir of the system is responsible for these processes (1 mT~30 MHz). Based on the results of the solution to the spin Hamiltonian (Equation (13)), it is possible to explain how the SR pulse forms based on quantum representations. Indeed, if we assume that the system’s pumping at RE implements the quick transitions of BC Co and BC Mn from the singlet state S to the excited singlet state S* and then to the excited triplet state T* (the process diagram for this is shown in Figure 12), once the spin Hamiltonian equation has been solved (Equation (13)), additional processes can be explained using the ESR data for BC Co and BC Mn. In fact, a splitting diagram of the triplet levels T_+1_, T_−1_ and T_0_ in a magnetic field can be created using the previous information for *J, G, D* and *E* (*J* and *G* are constants of isotropic and anisotropic exchange interaction, and *D* and *E* are constants of dipole–dipole and spin–spin interactions, respectively) (Figure 12).

At the same time, for two-spin systems with metal nuclei with *I* ≠ 0 (in our case, *I* = 9/2 and *I* = 5/2 for cobalt and manganese nuclear spins, respectively), the levels T_+1_ and T_−1_ are split due to the internal electric field *E*, and then each T_+1_ or T_−1_ level is split into a system of sublevels between which quantum transitions are allowed due to the removal of the degeneracy of the spin–spin interaction of electrons and nuclei under the influence of the internal magnetic field *H_int_* (it is known that *H_int_* is a function of the anisotropic exchange interaction—*H_int_* = *f*(*G*) [21,25]). Because of these interactions, T_+1_ and T_−1_ levels turn into wide bands (almost subzones—Figure 12). Within these subzones, electronic transition processes can take place, which are probably responsible for low-frequency bands in the range 0 to 100 MHz. The SR process is the realization of a forbidden transition between T_+1_ and T_−1_ levels, since only the splitting of *E* for BC Co and BC Mn equal to *E* = 5.83 (6.21) mT can be correlated and compared with the SR bands at 180 and 189.7 MHz (1 mT~30 MHz). The probable scheme of these processes is shown in Figure 12. The proposed scheme does not contradict the ideas of spin systems absorbing and emitting radio-frequency waves, which have been developing for a long time [74,75,76,77,78,79]. Thus, under the external influence of elastic pulses, the electron spin Zeeman reservoir formed by Dzyaloshinskii–Moriya dimers, i.e., a system of non-collinear spins, is inversely populated. This Zeeman spin reservoir is the source of the observed electromagnetic SR resulting from the annihilation of triplet excitations. The intensity of SR is directly related to the electronic properties and structure of two-spin systems, as well as the polymer matrix. The role of the polymer matrix is that the vibrations of the polymer chains lead to the activation of organometallic complexes. At the same time, it is reasonable to assume that mechanical activation occurs most actively in nano-objects with movable walls that occur during a RE in a polymer matrix of 2D nano-objects—plates with transverse dimensions of 50–100 nm and a thickness of ~1–2 nm (Figure 5a). [70]. These nanovolumes occur at the places of contact of the filler and polymer chains. At the same time, nanoparticles do not peel off from the matrix polymer under the influence of high pressure and shear deformation, which lead to RE [61]. In the complexes activated in this way, physico-chemical processes take place, including redox processes associated with redox-active ligands, with the transfer of electron density and the formation of mobile electrons. In this case, triplet excited states and negative spin polarization arise [76,77,78,79].

The BC Co and BC Mn complexes can be considered as spin catalysts of the superradiation process, representing a pair of unpaired electrons on metal atoms. Their occurrence is in good agreement with the concept of A.L. Buchachenko about the formation of a spin-selective nanoreactor due to the introduction of an oxygen atom along the M...O...M bond (where M is Co or Mn) with the formation of a triplet pair of radicals on metal atoms [80]. Recombination of this radical pair is stimulated by resonant microwave radiation at the Zeeman frequency at *g* = 2.0. This radiation forms a macrodipole, which implements the SR process—a SR pulse at characteristic frequencies for binuclear clusters.

This concept is also applicable for three-spin and four-spin systems. Indeed, in the works of A.L. Buchachenko [80,81], spin catalysis is considered for a three-spin system of the type (R_1_, R_2_, R_3_)—i.e., a radical pair and a third radical or paramagnetic ion, as well as for an isotropic four-spin system of the type (R_1_, R_2_, Ṁ)—i.e., a radical pair and a two-spin object. It is shown in [81] that when such systems are exposed to resonant microwave radiation at the Zeeman frequency at *g* = 2.0, the yields of products increase, i.e., the intensity of SR increases. However, the study of such effects is the subject of future research.

## 8. Conclusions

Experiments, investigating the processes of radio-frequency radiation and SR and subsequent analysis of the obtained radiation spectra simultaneously within the spin system model (Landau–Lifshitz and Bloch formulas) and dielectric dipoles system model (Havriliak–Negami and Lorentz methods) provide information about the processes of relaxation and transformation of the energy of elastic wave pulses into radio-frequency radiation and SR in various polymer composites, inorganic multiferroics and molecular magnets, and also analyze the earth’s crust in places of probable earthquakes. Accordingly, it is possible to create sources of pulsed radio-frequency SR based on polymer composites, inorganic multiferroics and molecular magnets. Moreover, following recent trends, work should be carried out in order to create devices operating in the range of short radio waves, i.e., in the terahertz range.

It is also possible to create molecular magnets for diagnostic and therapeutic purposes, due to radio-frequency radiation when the polarization and spin parameters change under influence of external alternating magnetic and electric fields. Such objects are purposefully delivered and diagnose and carry out therapeutic effects, and then, in the course of therapy, transform by the action of radio-frequency pulses into biologically harmless metal ions, antioxidants and polymers from which healthy tissue is formed. To achieve such goals, it is possible to use organoelement dimers of metals with ligands of spatially hindered phenols (antioxidants) incorporated into the polymer shell (for example, from polylysine or polylactic acid) that implement the Dzyaloshinskii–Moriya interaction. These objects can be an alternative to magnetic nanoparticles, which are currently widely used for medical purposes, since they are both a receiver and emitter of electromagnetic waves that carry out thermal and electromagnetic effects.

The reviewed methods can be used to analyze the earth’s crust in places of probable earthquakes, implementing mechanochemical control of the earthquake source. Indeed, the concept of energy storage as a cause of earthquakes by dislocations captured on stoppers has been intensively considered recently [80,81]. At the same time, it is postulated that the dislocation captured on the stopper is an electronic two-spin system that can exist for a very long time. The critical concentration of such objects results in an earthquake. Obviously, having a portable device for pulsed mechanical action, it is possible to monitor the earth’s crust for the accumulation of such “stabilized” dislocations.

It can also be assumed that the materials, implementing pulsed radio-frequency SR, are in demand for the creation of next-generation quantum computers. Indeed, their properties can be controlled by mechanical action, as well as by exposure to a magnetic or electric field in a contactless version.

## Figures and Tables

**Table 1:** Values of *ω*^2^*_R_* and α, as well as *ω*^2^*_pe3_* and *ω_r_* (*ω_H_*) obtained for the approximation of absorption lines by the Lorentz method and by the LLB method. The definition of the coupling coefficient *k* between these values is given in the text (From [48] with permission).

The Lorentz Equation	*ω* ^2^ *_R_*	*α*	*k*
*PS*	1500	30	1.67
	9900	29.1	0.6
*PS*–BC Co	350	15	1.60
	2450	15	0.61
	32,300	6	0.06
*PS*–BC Mn	450	10.5	0.99
	2200	16	0.68
	36,500	12.5	0.13
LLB equation	*ω* ^2^ *_pe3_*	*ω_r_* (*ω_H_*)	*k*
*PS*–BC Co	130	19.5 (14.9)	3.42 (2.61)
	2190	22.1 (21.5)	0.94 (0.91)
	32,300	10.0 (9.37)	0.11 (0.10)
*PS*–BC Mn	225	14.0(14.0)	1.86 (1.86)
	2350	20.4(20.9)	0.84 (0.86)
	36,100	27.6(26.4)	0.29 (0.27)

**Table 2:** Values of the coefficients *logf*_0_, Δ*ε*, *a* and *b* for the Havriliak–Negami model, which allow for the construction of curves of the distribution of relaxation times *g(τ)* for bands with numbers given in parentheses and correspond to the numbers in Figure 8 (From [48] with permission).

***PS*** Bands	*logf*_0_	Δ*ε*	*a*	*b*	*τ_max_*, ns
23.98 MHz (1)	8.41	14.30	0.82	9.26	19.6
97.47 MHz (2)	7.97	2.65	1.79	0.76	9.4
***PS*** + Co(QH)_2_ bands					
12.1 MHz (1)	8.08	1.41	0.89	11.46	61
46.9 MHz (2)	7.71	0.41	1.57	1.40	63
180.1 MHz (3)	8.25	0.018	1.95	0.96	5
***PS*** + Mn(QH)_2_ bands					
16.9 MHz (1)	7.38	1.017	1.23	1.23	41
49.3 MHz (2)	7.70	0.10	1.66	1.11	18
189.7 MHz (3)	8.28	0.017	1.920	1.04	5

**Table 3:** Parameters of the radiation bands.

	P1	P2	P3	P4	P5	P6	P7	P8
ω_res,E_, MHz	9.5	27.0	50.0	75.0	175.0	204.0	255.0	306.0
ω_r,E_, MHz	5.0	10.0	10.0	10.0	10.0	10.0	10.0	10.0
ω_C_, MHz	8.1	25.1	49.0	71.3	174.7	203.8	254.8	305.8
ε_0_	4061	1588.5	596.9	320.0	31.7	29.1	10.0	4.7
ω_res,H_, MHz	9.5	27.0	50.0	75.0	175.0	204.0	255.0	306.0
ω_r,H_, MHz	5.0	10.0	10.0	10.0	10.0	10.0	10.0	10.0
ω_H_, MHz	8.1	25.1	49.0	71.3	174.7	203.8	254.8	305.8
χ_0_	4625	1389	549.0	279.3	31.8	35.0	12.91	4.6

**Table 4:** Parameters of the Fourier bands of the spectrum for the SR bands for K5 and those of the Fourier bands of the PS spectrum (Havriliak–Negami model).

The Band of SI Spectrum	*log*ω_0_	Δ*ε*	*a*	*b*	*τ*_0_, ns
**P1**	7.616	1.289	1.235	1.320	28
**P2**	8.202	0.366	1.631	1.025	5.2
**P3**	8.488	0.172	1.777	1.005	3.0
**P4**	8.668	0.096	1.844	1.002	2.3
**P5**	9.039	0.0077	1.930	1.000	0.9
**P6**	9.106	0.0073	1.940	1.000	0.8
**P7**	9.203	0.0025	1.951	1.000	0.6
**P8**	9.286	0.0011	1.959	1.000	0.5
**PS_1**	8.295	12.331	0.919	8.471	12.0
**PS_2**	8.032	2.229	1.576	1.579	27.0

*τ*_0_, ns—coincides with the maxima of the relaxation curves in Figure 11d (the calculation of the relaxation curves was carried out according to Equation (6) and data from Table 4).

## Data Availability

Not applicable.
